# isoespy: an integrated long-read transcriptome workflow for isoform resolution and visualization

**DOI:** 10.1093/bioadv/vbag044

**Published:** 2026-02-13

**Authors:** Ko Ikemoto, Akihiro Fujimoto

**Affiliations:** Department of Human Genetics, Graduate School of Medicine, The University of Tokyo, Tokyo 113-0033, Japan; Department of Human Genetics, Graduate School of Medicine, The University of Tokyo, Tokyo 113-0033, Japan

## Abstract

**Summary:**

Long-read RNA-seq uncovers complex transcriptome diversity, opening new avenues for isoform-level expression analysis. Nevertheless, the functional diversity of individual isoforms is still poorly understood. We introduce isoespy, an analysis pipeline for integrating isoform structures, differential expression, and functional annotations from long-read RNA-seq data. The workflow integrates third-party open reading frame predictors, juxtaposes isoform expression levels with gene models, and visualizes positional and non-positional user-provided features. We applied isoespy to a transcriptome dataset of hepatocellular carcinoma, identifying differences in isoform usage and predicted protein function. isoespy facilitates the interpretation of transcriptomic complexity through integrated structural and functional visualization.

**Availability and implementation:**

Isoespy is freely available at https://github.com/kolikem/isoespy.

## 1 Introduction

Transcript isoforms, which are RNA molecules that differ in sequence despite sharing a gene locus, are key determinants of cellular state. Recent technological advances in long-read RNA sequencing (RNA-seq) allow for the direct capture of full-length transcripts, enabling unprecedented accuracy in isoform-resolved analysis (Monzó *et al.* 2025). Such analysis has revealed cancer-specific and tissue-specific isoforms that remain hidden at the gene level and unveiled numerous novel transcripts (Sun *et al.* 2023, Shimada *et al.* 2024). However, few pipelines unite isoform-level differential analyses with intuitive visualization. Several visualization libraries are currently available (Table 1). For instance, Gviz (Hahne and Ivanek 2016) and ggtranscript (Gustavsson *et al.* 2022) offer high flexibility and customizability for genomic data visualization. However, these tools generally operate as low-level plotting libraries, typically requiring users to possess advanced coding skills to construct transcript structures from dataframes. IsoformSwitchAnalyzeR (Vitting-Seerup and Sandelin 2019) is a comprehensive R suite that enables detailed analysis of isoform switches. Although powerful, its framework is primarily designed for differential isoform usage (DIU) analysis; therefore, transcript-level differential expression (DE) analysis and its structural interpretation are not fully integrated into its standard workflow. Swan (Reese and Mortazavi 2021) is designed for the interpretation of long-read data and includes both DE and DIU analyses. While effective for graph-based interpretation, its framework does not implement functionality to visualize functional features on transcript models alongside gene models and statistical results in an integrated manner. Other tools, such as IsoTV (Annaldasula *et al.* 2021) and IsoVis (Wan *et al.* 2024), are primarily designed for exploratory tasks rather than offering a streamlined pipeline that integrates statistical differential analysis. Consequently, there is a need for an end-to-end framework that complements these existing tools by focusing on the automation of DE and DIU analyses and the integrated visualization of statistical results, gene models, and functional features.

**Table 1:** Comparison of key features between isoespy and related tools.

	isoespy	Gviz	ggtranscript	IsoformSwitchAnalyzeR	Swan	IsoTV	IsoVis
Primary design	Python analysis suite	R visualization library	R visualization library	R analysis suite	Python analysis suite	Python analysis suite	Javascript application
DE analysis and visualization	Yes	Limited	Limited	Limited	Yes	Limited	Limited
DIU analysis and visualization	Yes	Limited	Limited	Yes	Yes	Limited	Limited
Functional feature visualization	Yes	Yes	Yes	Yes	Limited	Yes	Yes
Gene model visualization	Yes	Yes	Yes	Yes	Limited	Yes	Yes

Here, we introduce isoespy (isoform expression and structure analysis pipeline), a Python toolkit that draws transcript structures, juxtaposes expression levels in two conditions based on DE and DIU analyses, and overlays functional annotations such as protein domain through a streamlined series of Python commands. A feature comparison to other tools is provided in Table 1. Requiring only a transcript annotation file in the gene transfer format (GTF) and a matching count matrix, isoespy delivers seamless analysis and interpretable figures. We illustrate its performance on an Oxford Nanopore (ONT) long-read RNA-seq dataset comprising 42 pairs of hepatocellular carcinomas (HCCs) and matched nontumor liver samples (Kiyose *et al.* 2022).

## 2 Implementation

### 2.1 Overview of isoespy

**Figure 1:**
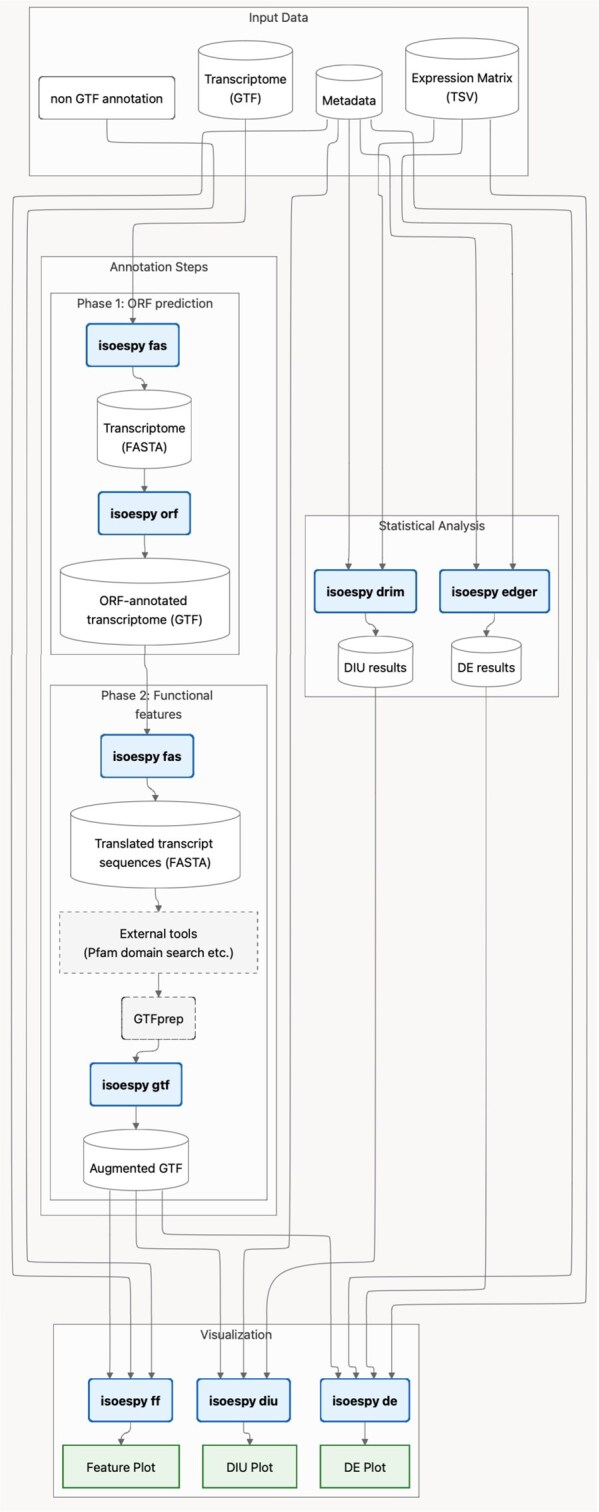
Schematic overview of the isoespy workflow. The workflow takes a transcriptome annotation in GTF and expression count matrix as primary inputs. Using external tools, it performs (i) ORF prediction for novel transcripts, (ii) functional feature prediction, (iii) DE analysis, and (iv) DIU analysis. Based on these analyses, isoespy provides three corresponding visualization methods: *isoespy ff*, *isoespy de*, and *isoespy diu*.

isoespy is a Python suite for two-group transcriptome comparisons. Required inputs include (i) a transcriptome in GTF and (ii) matching expression data in raw counts (Fig. 1). The four independent modules, *isoespy orf* for open reading frame (ORF) prediction, *isoespy de* for DE visualization, *isoespy diu* for DIU visualization, and *isoespy ff* for functional feature plotting, can be executed separately or chained.

### 2.2 Analysis workflow and visualization

*isoespy orf* is a module that predicts the ORFs of novel transcripts lacking coding sequence (CDS) annotations in the original GTF file. Given a GTF, *isoespy orf* appends ORF annotations by scoring the coding potential with CPAT (Wang *et al.* 2013) and selecting the most likely frame with TransDecoder (Haas *et al.* 2013). This step can be performed prior to visualizations, and the augmented GTF is passed downstream. *isoespy edger* takes as input a GTF file and a count matrix, runs edgeR (Robinson *et al.* 2010) with a two-group design, and then prepares intermediate files for the DE visualization. *isoespy de* combines the DE results with the GTF file, thus enabling visualization of the DE status for isoforms of a gene with its genomic structure. *isoespy drim* performs DIU analysis and generates the necessary output files for visualization by *isoespy diu*. It leverages the external tool DRIMSeq (Nowicka and Robinson 2016) to test for isoform usage within genes, followed by stageR (Van den Berge et al. 2017) for multiple testing correction to control the overall false discovery rate (FDR). Subsequently, the *isoespy diu* command visualizes these results. *isoespy de* and *isoespy diu* are tool-agnostic, so users can visualize results generated by external tools other than edgeR and DRIMSeq as long as the input format is compatible. *isoespy ff* visualizes both positional features (e.g. protein domain, signal sequences) and non-positional attributes [e.g. subcellular localization, expression level, and nonsense-mediated decay (NMD) status]. Non-positional features are classified as binary, categorical, or continuous and can be displayed alongside positional features.

### 2.3 Application to liver cancer transcriptome

In order to assess the workflow, we applied isoespy to an ONT cDNA sequencing dataset derived from HCCs and matched paracancerous liver tissues from 42 patients (Table 1, available as supplementary data at *Bioinformatics Advances* online, Kiyose *et al.* 2022). Transcripts were identified and quantified using SPLICE (Kiyose *et al.* 2022) with GENCODE v44 as the reference. Unannotated isoforms were processed with *isoespy orf*. The DE analysis was performed using *isoespy edger* on raw counts, applying trimmed mean of M values (TMM) normalization and the quasi-likelihood (QL) F-test. The DIU analysis was performed using *isoespy drim* with the likelihood ratio test (LRT), followed by stageR for stage-wise FDR control. In both analyses, statistical significance was defined as an adjusted *P* value <.05. Protein features were predicted with Pfam-A v37 (Mistry *et al.* 2021) and HMMER v3.4 (Eddy 2011) for the protein domain, SignalP v6.0h (Teufel *et al.* 2022) for the signal peptide, and NLStradamus v1.8 (Nguyen Ba et al. 2009) for the nuclear localization signal (NLS). The results of these functional predictions were integrated into a GTF with functional annotations, which served as the input for visualizations using *isoespy de*, *isoespy diu*, and *isoespy ff*.

## 3 Results

**Figure 2:**
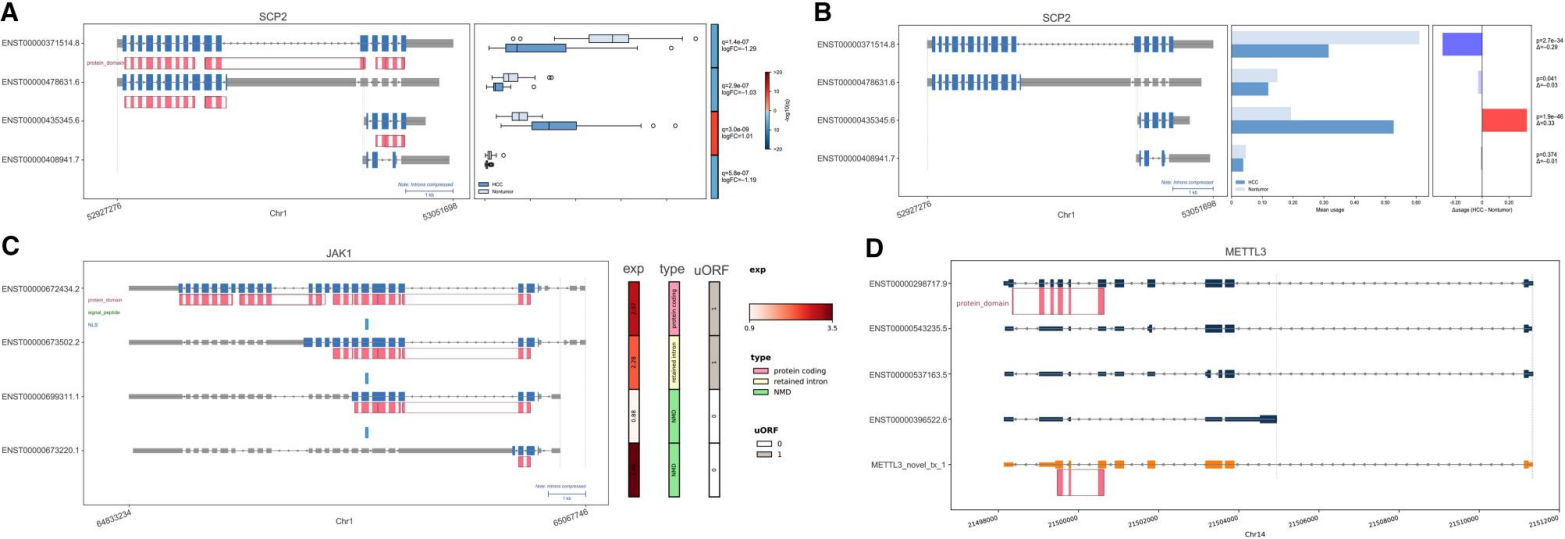
Transcript isoform structures, expression levels, and annotation features visualized for several genes. (A) Visualization of DE analysis for *SCP2*. Expression levels in nontumor and HCC samples are displayed as box plots for each isoform. The *q*-values and log2 fold changes are displayed on the right panel. The significance threshold was set at adjusted *P* value <.05 (QL F-test). Auxiliary lines indicate the transcription start sites (TSSs). Introns are compressed to 100 bp. (B) Visualization of DIU analysis for *SCP2*. Isoform usage in nontumor and HCC samples is shown as bar plots. In the right panel, the usage in case samples relative to controls is visualized using bar plots, annotated with *P* values and Δusage. The significance threshold was set at adjusted *P* value <.05 (LRT). Auxiliary lines indicate the TSSs. Introns are compressed to 100 bp. (C) Visualization of the functional features of *JAK1*. Tracks for protein domains, signal peptides, and NLS are displayed. The expression level (continuous data), biotype (categorical data), and uORF presence (binary data) are presented on the right. Auxiliary lines indicate the TSSs. Introns are compressed to 100 bp. (D) Visualization of the *METTL3* gene model. The detected novel isoform (METTL3_novel_tx_1) shares a TSS with known isoforms. ORF prediction identified the same protein domains as the dominant isoform (ENST00000298717.9).

We analyzed the HCC dataset using isoespy, based on 53 219 transcripts identified and quantified by SPLICE software. Here, we highlight several representative genes to demonstrate distinct regulatory patterns. The sterol carrier protein 2 gene (*SCP2*) produced four isoforms (Fig. 2A and B). Although the gene-level expression was downregulated in HCC, isoform-level analysis using isoespy revealed a more complex regulation. Three isoforms showed downregulation in HCCs (mean log_2_FC = –1.17, FDR < 0.01), whereas the other (ENST00000435345.6) was conversely upregulated (log_2_FC = 1.01), indicating differential isoform usage for this gene (Fig. 2A). Indeed, the DIU analysis revealed that ENST00000435345.6 exhibited a 33% increase in usage (*P* = 1.9 × 10^−46^) (Fig. 2B). Interestingly, while ENST00000478631.6 and ENST00000408941.7 showed only marginal changes in usage, ENST00000371514.8 displayed a 29% decrease in usage (*P* = 2.7 × 10^−34^). This suggests that the reduction in ENST00000371514.8 was almost entirely compensated for by the increase in ENST00000435345.6. Structurally, the upregulated isoform retains the SCP2 domain (PF02036) but lacks other domains present in the downregulated isoform (ENST00000371514.8), suggesting distinct functional consequences in HCC.

In contrast, the Janus kinase 1 gene (*JAK1*) harbored four isoforms with no significant difference in overall gene expression between HCC and nontumor groups (Fig. 2C). One isoform (ENST00000673220.1), annotated to undergo NMD, was dominant in both conditions. The gene employed alternative transcription start sites (TSSs). Two isoforms (ENST00000699311.1 and ENST00000673220.1) initiated from a proximal TSS and the others (ENST00000673502.2 and ENST00000672434.2) from a distal TSS. Notably, we found that the TSS usage was linked to NMD susceptibility and predicted upstream open reading frame (uORF) presence (Fig. 2C). In addition to characterizing known isoforms, we explored isoespy’s capability to visualize novel isoforms in a biologically interpretable manner using the methyltransferase 3, N6-adenosine-methyltransferase complex catalytic subunit gene (*METTL3*). We identified a novel transcript (METTL3_novel_tx_1) that is structurally distinct from the annotated isoforms (Fig. 2D). The visualization revealed that while the novel isoform retains the key protein domain (PF05063) comparable to the canonical isoform ENST00000298717.9, it exhibits earlier transcription termination. This structural difference suggests that, despite retaining the coding domain, the transcript might be a target for NMD or possess distinct mRNA stability compared to the full-length isoform. Overall, isoespy enables the integrated analysis of gene isoform structures, functional features, and isoform-level differential analyses between two groups. Therefore, this analysis pipeline can contribute to explorations of the biological significance of gene isoforms in various diseases and traits.

## Supplementary Material

vbag044_Supplementary_Data

## Data Availability

Publicly available datasets were analyzed in this study. The ONT cDNA sequencing data derived from HCC and matched paracancerous liver tissues (Kiyose *et al.* 2022) are available in the Japanese Genotype-phenotype Archive (JGA) under accession number JGAD000635. The sample-level metadata necessary to reproduce the analysis are available at https://github.com/kolikem/isoespy.
